# Differences in shuntflow (Qa), cardiac function and mortality between hemodialysis patients with a lower-arm fistula, an upper-arm fistula, and an arteriovenous graft

**DOI:** 10.1177/11297298221092741

**Published:** 2022-04-23

**Authors:** Johannes W Drouven, Janke Wiegersma, Solmaz Assa, Adrian Post, Mostafa El Moumni, Akin Özyilmaz, Clark J Zeebregts, Casper FM Franssen

**Affiliations:** 1 Division of Vascular Surgery, Department of Surgery, University Medical Center Groningen, University of Groningen, Groningen, The Netherlands; 2 Division of Nephrology, Department of Internal Medicine, University Medical Centre Groningen, Groningen, The Netherlands

**Keywords:** Dialysis, dialysis access, AV fistula, prosthetic grafts, economics and health services, catheters

## Abstract

**Background:**

High-flow vascular accesses may contribute to cardiovascular morbidity and mortality in hemodialysis patients. Since shuntflow (Qa) varies between vascular access types, the current study aims to investigate differences in left ventricular hypertrophy (LVH), systolic and diastolic function parameters, and all-cause mortality between patients with a lower-arm arteriovenous fistula (AVF), an upper-arm AVF, and an arteriovenous graft (AVG).

**Methods:**

A post hoc analysis of 100 patients was performed in a single-center, prospective observational study. Echocardiography examinations were performed prior to the dialysis session. Qa measurements were performed using ultrasound dilution. Patient groups were categorized by vascular access type. Cox proportional hazards models were used to investigate the association of shunt type with all-cause mortality with adjustment for potential confounders including, amongst others, age, sex, diabetes, the duration of hemodialysis treatment, shunt vintage, and Qa.

**Results:**

Patients with an upper-arm AVF had significantly (*p* < 0.001) higher Qa (median 1902, IQR 1223–2508 ml/min) compared to patients with a lower-arm AVF (median 891, IQR 696–1414 ml/min) and patients with an AVG (median 881, IQR 580–1157 ml/min). The proportion of patients with LVH and systolic and diastolic echocardiographic parameters did not differ significantly between groups. Survival analysis showed that an upper-arm AVF was associated with a significantly lower all-cause mortality (*p* = 0.04) compared to a lower-arm AVF.

**Conclusions:**

Patients with an upper-arm fistula had a higher Qa but similar systolic and diastolic cardiac function. Patients with an upper-arm fistula had a significantly lower risk of all-cause mortality compared with patients with a lower-arm fistula.

## Introduction

Worldwide, the number of patients with end-stage renal disease requiring intermittent hemodialysis is increasing.^1,2^ A vascular access with sufficient flow is mandatory to perform adequate hemodialysis. A radiocephalic arteriovenous fistula (AVF) is considered the access of first choice in most patients.^2^

Hemodialysis patients have markedly increased cardiovascular morbidity and mortality, with left ventricular hypertrophy (LVH) and left ventricular systolic and diastolic dysfunction as the most common cardiac abnormalities.^3,4^ The cause of cardiac complications is multifactorial with conventional risk factors having a modest contribution whereas fluid overload, bone and mineral disorders, chronic inflammation, endothelial dysfunction, and hemodialysis treatment itself are considered to have a more important role.^4–11^ There is increasing awareness that a vascular access may contribute to cardiac complications. This link is most obvious in patients with prolonged high vascular access flow (Qa) that have an increased risk to develop high-output cardiac failure.^4–9^ Basile et al. were one of the first to show that higher Qa are strongly associated with a higher cardiac output,^6^ although the exact pathophysiology behind this process has not been fully elucidated.^12^

Since the Qa may vary between different types of vascular access, the effect on the heart may also differ between different types of vascular access. It can be hypothesized that the vascular access type with the highest Qa, that is the brachiocephalic AVF, may have a negative effect cardiac function and that patients with such an access are particularly prone to the development or worsening of cardiac failure.^8,13–19^ However, literature in which echocardiographic parameters and long-term outcomes are compared between patients with different types of arteriovenous access is absent.

The primary objective of this study was to determine whether there are differences in left ventricular hypertrophy (LVH), systolic and diastolic cardiac function parameters between patients with a lower-arm AVF, an upper-arm AVF, and an arteriovenous graft (AVG). The secondary objective was to determine if there is an association between the type of vascular access and all-cause mortality.

## Materials and methods

### Study design

The current study is a post hoc analysis of a single-center, prospective observational study that was originally designed to assess the prevalence and prognostic value of hemodialysis-induced regional left ventricular dysfunction in hemodialysis patients.^20^ Hemodialysis patients from the Dialysis Center Groningen and the University Medical Center Groningen were considered eligible for inclusion in our study if they were treated with hemodialysis for more than 3 months and were on a thrice-weekly hemodialysis schedule. Of the 235 in-center hemodialysis patients that were screened for potential inclusion in the original study, 76 patients did not meet the inclusion criteria and/or had exclusion criteria for this study. Of these, 27 patients were excluded because of NYHA stage IV heart failure. For this analysis, patients with a central venous catheter (*n* = 9) as hemodialysis access were excluded.

The study was performed according to the Declaration of Helsinki and was approved by the medical ethics committee of the University Medical Center Groningen (METc: 2008/343). All patients signed written informed consent. Echocardiography examinations were performed between March 2009 and March 2010. Mortality data was recorded until January 2015. There was no loss to follow-up.

### Creation of arteriovenous access

Vascular access creation was scheduled approximately 3 months before the expected start of hemodialysis in patients receiving an AVF, and 4–6 weeks in case of an AVG. Following the KDOQI and national guidelines, a fistula first approach was used in vascular access creation.^1^ In case of patients with previous maturation problems, or patients with a subacute indication for dialysis (i.e. <4 weeks), an AVG was considered instead of the fistula first approach. In AVG patients, a standard wall polytetrafluorethylene (PTFE) graft (Gore-Tex, WL Gore & Associates, Flagstaff, Arizona, USA) with 6 mm diameter and 0.5 mm wall thickness was used, in either a loop or straight configuration in the lower-arm or upper-arm. Lower-arm AVF patients received a radiocephalic AVF. Upper-arm AVF patients received a brachiocephalic AVF or basilic vein transposition.

### Echocardiography examination

Patients were studied just before the dialysis session after the longest interdialytic interval. All examinations were performed on the dialysis unit and were conducted by a team of three experienced technicians. Two-dimensional echocardiography was performed, including color flow mapping, and tissue Doppler echocardiography. All analyses were performed off-line according to the guidelines of the European Society of Echocardiography.^21^ At least three consecutive heartbeats in each view were acquired. Global systolic function was evaluated by left ventricular ejection fraction (LVEF) calculated using the biplane Simpson’s method. Left ventricle mass index (LVMi) was calculated as described previously.^22^ Left ventricular hypertrophy (LVH) was defined as LVMi > 95 g/m^2^ for women and >115 g/m^2^ for men.^23^ Peak early (*E*) and late (*A*) diastolic filling velocities, deceleration time, and isovolemic relaxation time were measured. Mean *eʹ* and *Sʹ* were derived from tissue Doppler early diastolic and peak systolic velocity respectively, on the lateral, septal, anterior, and inferior junctions of the myocardium and mitral valve annulus. The average *eʹ* (mean *eʹ*) and *Sʹ* (mean *Sʹ*) values were calculated from these values.

### Qa measurements

Ultrasound dilution flow measurements were performed with a Transonic HD01 plus Hemodialysis Monitor (Transonic Systems Inc., Ithaca, NY, USA). Qa was measured at 3-monthly intervals as part of routine patient care. For this study we used the average flow of the measurements before and after the dialysis session at which the echocardiographic examination was performed.

### Statistical analysis

Statistical analyses were performed using SPSS 24 (SPSS, Chicago, IL, USA) and R version 3.6.2 software (The R-Foundation for Statistical Computing). A two-sided *p* value less than 0.05 was considered to indicate statistical significance. Data is presented as mean with standard deviation (SD) for continuous variables with normal distribution, or as median and interquartile range (IQR) for skewed variables. Assessment of normality was tested with the Kolmogorov–Smirnov test. Patient groups were compared based on the type of vascular access.

Differences between groups were tested using the Pearson Chi-Square test and Kruskall–Wallis test for categorical data and non-normally distributed data, respectively. Correlation coefficients were calculated using the Pearson’s *R* test.

Prospective analyses of vascular access with transplantation-censored all-cause mortality were performed using Cox proportional hazard models. The proportional hazards assumption was verified visually with plots of the scaled Schoenfeld residuals and was not violated in any of the models. To account for differences in the characteristics between patients with different types of shunts, adjustments were made for the following variables: age, sex, body mass index (BMI), hemodialysis duration, diabetes mellitus, history of cardiovascular disease, hypertension, angiotensin receptor blockage use, shunt vintage (the time since creation of the vascular access), and Qa. To account for potential bias that could result from the exclusion of participants with missing values,^24^ multiple imputation using Fully Conditional Specification was performed to obtain five imputed data sets, in which Rubin’s rules were applied to acquire pooled estimates of the regression coefficients and their standard errors across the imputed data sets.^25^

To visualize the association of the type of vascular access with transplantation-censored all-cause mortality, a Kaplan–Meier curve was plotted.

## Results

### Patient characteristics

A total of 109 patients participated in the original study.^10^ Nine of these patients had a central venous catheter as vascular access and were excluded for this study. The characteristics of the remaining 100 patients categorized per access type are shown in Table 1. Hypertension (18%), autosomal dominant polycystic kidney disease (12%), and diabetes mellitus (12%) were the major causes of renal failure. Except for a difference in the proportion of males and the time on dialysis, no other significant differences between groups were observed. The time on dialysis was significantly longer in patients with a PTFE AVG compared with those with a lower-arm AVF or upper-arm AVF (50.9 (IQR 26.8–69.8), 19.1 (IQR 8.0–49.8), and 25.1 (IQR 13.1–47.9) months, respectively; *p* = 0.024).

**Table 1.** Patient characteristics and shuntflow.

	Lower-arm AVF	Upper-arm AVF	PTFE AVG	*p*-Value
Number of patients	46	32	22	
Age (y)	65.6 (52.4–74.8)	58.5 (49.1–68.9)	71.9 (59.3–79.0)	0.074
Male	36 (78%)	22 (69%)	10 (45%)	0.025
Body mass index (kg/m^2^)	26.1 (22.9–28.5)	25.4 (23.2–27.7)	26.7 (23.0–30.4)	0.464
Time on dialysis (mo)	19.1 (8.0–49.8)	25.1 (13.1–47.9)	50.9 (26.8–69.8)	0.024
**Shunt vintage**	**17.1 (8.0–42.4)**	**22.8 (13.1–45.8)**	**45.0 (26.8–54.0)**	**0.065**
Diabetes mellitus	12 (26%)	4 (13%)	6 (27%)	0.288
Hypertension	40 (87%)	23 (72%)	18 (82%)	0.246
Cardiovascular history	11 (24%)	6 (19%)	5 (23%)	0.860
Systolic blood pressure (mmHg)	137 (29)	132 (32)	143 (30)	0.567
Diastolic blood pressure (mmHg)	78 (16)	80 (14)	75 (23)	0.894
Heart rate (bpm)	73 (18)	75 (18)	64 (13)	0.067
Medication				
Angiotensin receptor blocker	4 (9%)	8 (25%)	1 (5%)	0.051
Aspirin	22 (48%)	16 (50%)	17 (77%)	0.076
Beta blocker	28 (61%)	16 (50%)	12 (55%)	0.628
Calcium channel blockers	7 (15%)	3 (9%)	3 (14%)	0.708
Diuretics	3 (7%)	2 (6%)	1 (5%)	0.936
Qa (ml/min)	891 (696–1414)	1902 (1223–2508)	881 (580–1157)	<0.001

Data are presented as median (IQR) or number (%).

AVF: arteriovenous fistula; AVG: arteriovenous graft; PTFE: polytetrafluoroethylene.

### Vascular access flow (Qa)

Patients with an upper-arm AVF had a significantly higher Qa (1902 (IQR 1223–2508) ml/min) compared to patients with a lower-arm AVF (891 (IQR 696–1414) ml/min) or compared to those with a PTFE AVG (881 (IQR 580–1157) ml/min), *p* < 0.001.

### Echocardiographic data, systolic and diastolic function parameters

LVMi and the proportion of patients with LVH did not differ significantly between the three groups (Table 2). Five patients in the lower-arm AVF group had significant (grades 2–3) mitral valve insufficiency versus three in the upper-arm AVF group and one in the PTFE AVG group. No significant differences were found in the systolic and diastolic echocardiographic parameters between groups.

**Table 2.** Echocardiographic parameters.

	Lower-arm AVF	Upper-arm AVF	PTFE AVG	*p*-Value
Number of patients	46	32	22	
Systolic function				
LVEF	52.5 (42.5–58.0)	52.0 (46.5–57.0)	49 (40.0–54.5)	0.518
Mean *S*’	5.48 (3.94–6.67)	5.73 (4.42–7.26)	4.69 (3.96–5.44)	0.174
Diastolic function				
*E* (m/s)	0.90 (0.82–1.11)	0.93 (0.81–1.11)	0.97 (0.72–1.06)	0.749
*A* (m/s)	0.81 (0.66–0.97)	0.90 (0.74–1.05)	0.82 (0.77–0.98)	0.417
*E*/*A*	1.06 (0.92–1.33)	1.09 (0.91–1.37)	1.07 (0.77–1.22)	0.747
DT (ms)	200 (163–240)	215 (192–238)	219 (180–277)	0.482
IVRT (ms)	81 (68–113)	94 (74–113)	100 (85–105)	0.248
Mean *e*ʹ (cm/s)	7.3 (5.1–8.0)	6.4 (5.2–8.7)	5.6 (4.5–6.6)	0.081
*E*/*e*ʹ	13.3 (11.2–17.3)	14.8 (10.5–18.3)	16.2 (12.8–17.2)	0.254
Left ventricular mass				
LVMi (g/m^2^)	93.6 (74.2–111.2)	93.1 (75.8–110.4)	95.1 (71.6–112.8)	0.994
LVH	9 (21%)	8 (25%)	6 (27%)	0.174

Data are presented as median (IQR) or number (%).

*A*: peak velocity of late transmitral flow; *E*: peak velocity of early diastolic transmitral flow; DT: deceleration time; *eʹ*, peak velocity of early diastolic mitral annular motion as determined by tissue Doppler; *E*/*eʹ, E* to *eʹ* ratio; IVRT: isovolemic relaxation time; LVEF, left ventricular ejection fraction; LVH: left ventricular hypertrophy; LVMi, left ventricular mass index; *Sʹ*, peak systolic velocity of mitral annular motion as determined by tissue Doppler.

### Survival analysis

During a mean follow-up of 4.8 (±3.1) years, 46 (46%) patients died. Cox regression analyses of the association between the type of vascular access and transplantation-censored all-cause mortality are shown in Table 3. Compared to patients with a lower-arm AVF, an upper-arm AVF was associated with a lower risk of all-cause mortality in the crude analysis (HR [95% CI]: 0.24 [0.10–0.58]; *p* = 0.002). This association remained significant after adjustment for age, sex, BMI, dialysis vintage, diabetes mellitus, history of cardiovascular disease, hypertension, angiotensin receptor blocker usage, and shunt vintage (model 5, *p* = 0.01). After additional adjustment for Qa, patients with an upper-arm AVF still had a lower all-cause mortality compared with patients with a lower-arm AVF (model 6, *p* = 0.04). There was no significant difference in all-cause mortality between patients with a lower-arm AVF and those with a PTFE AVG, nor between patients with an upper-arm AVF and a PTFE AVG.

**Table 3.** Prospective analyses of vascular access on transplantation-censored all-cause mortality.

Model	Type of vascular access				
	Lower-arm AVF	Upper-arm AVF		PTFE AVG	
	HR (95% CI)	HR (95% CI)	*p*-Value	HR (95% CI)	*p*-Value
1	Ref (1.00)	0.24 [0.10–0.58]	0.002	0.59 [0.29–1.24]	0.16
2	Ref (1.00)	0.29 [0.12–0.71]	0.008	0.70 [0.32–1.56]	0.38
3	Ref (1.00)	0.29 [0.12–0.70]	0.008	0.83 [0.35–1.99]	0.67
4	Ref (1.00)	0.31 [0.12–0.77]	0.01	0.66 [0.29–1.48]	0.30
5	Ref (1.00)	0.30 [0.12–0.73]	0.01	0.71 [0.32–1.59]	0.39
6	Ref (1.00)	0.38 [0.15–0.96]	0.04	0.60 [0.26–1.35]	0.21

AVF: Arteriovenous fistula; PTFE: polytetrafluoroethylene; AVG: Arteriovenous graft.

Model 1: Crude; Model 2: Adjusted for age and sex; Model 3: As model 2, additionally adjusted for BMI and hemodialysis vintage; Model 4: As model 2, additionally adjusted for diabetes mellitus, history of cardiovascular disease, angiotensin receptor blocker usage, and hypertension. Model 5: As model 2, additionally adjusted for shunt vintage. Model 6: As model 2, additionally adjusted for Qa.

Additional survival analysis between the type of vascular access and cardiovascular mortality showed no significant association, data not shown.

**Figure 1.**
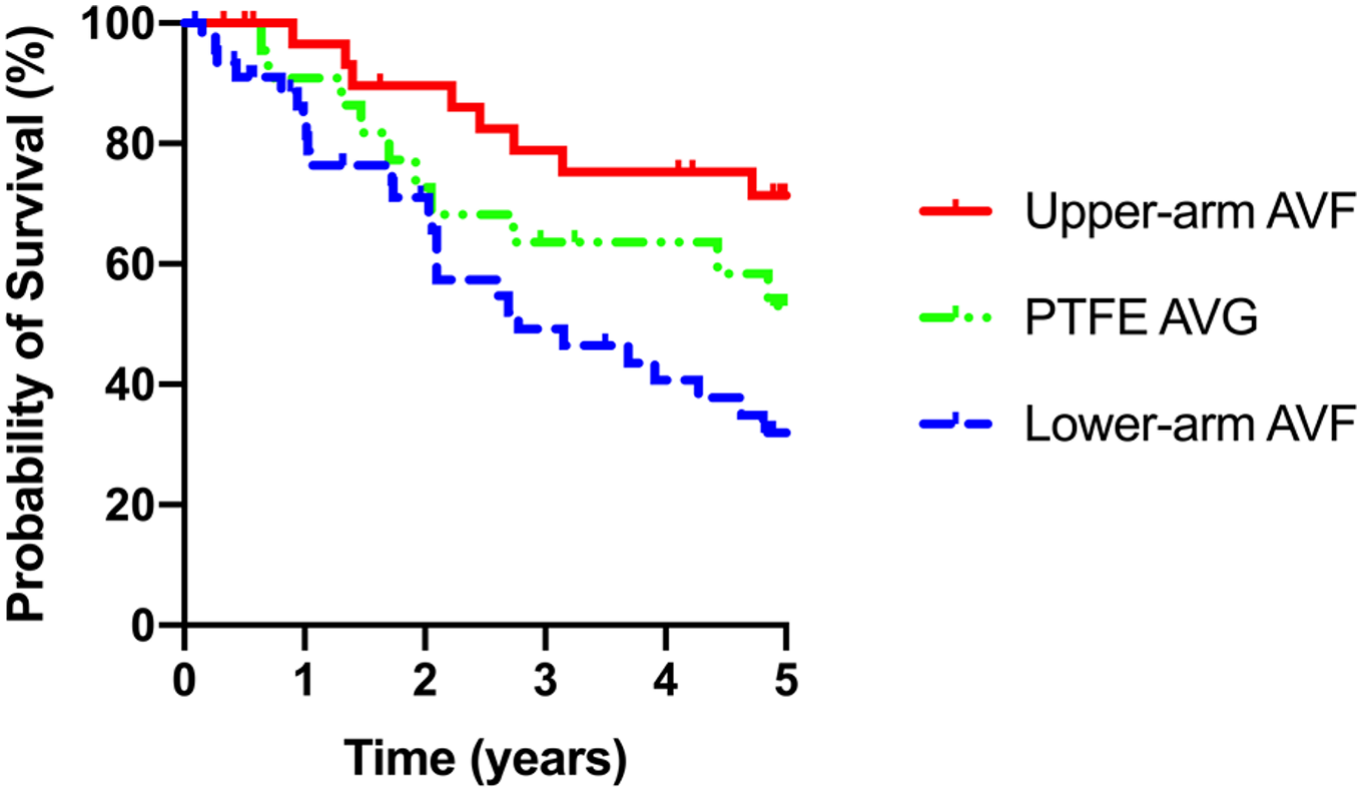
Survival analysis. Kaplan–Meier curve of transplantation-censored all-cause mortality according to the type of vascular access (Log-rank test *p*-value = 0.002).

A Kaplan–Meier curve of the association between the type of vascular access and all-cause mortality is shown in Figure 1.

## Discussion

In this study, our primary objective was to evaluate whether there are differences in left ventricular hypertrophy (LVH), and systolic and diastolic cardiac function parameters between different types of vascular access. Our secondary objective was to determine if there is an association between the type of vascular access and all-cause mortality. Interestingly, systolic and diastolic function parameters did not differ significantly between the types of vascular access in spite of a significantly higher Qa in patients with an upper-arm AVF, compared to patients with a lower-arm AVF and those with a PTFE AVG. The access type with the highest Qa, the upper-arm AVF, was independently associated with a lower all-cause mortality. Patients with an upper-arm AVF had a significantly better survival compared with those with a lower-arm AVF, even after correction for potential confounders including age, sex, diabetes, duration of dialysis treatment, and shunt vintage. The significant longer time on dialysis in patients with an AVG might be explained by the vascular access strategy in our center. An AVG is only used when previous autologous vascular accesses failed or were not possible.

Several studies analyzed the association between Qa and cardiac function and cardiac failure in vascular access patients. Pandeya et al. found a linear relationship between Qa and cardiac output in 16 patients on hemodialysis, of which 11 patients had a lower-arm AVF and 5 patients a PTFE AVG.^12^ Basile et al. investigated the relationship between Qa, cardiac output, and cardiac failure in a group consisting of 65 patients with a lower-arm AVF and 31 patients with an upper-arm AVF.^6^ Ten patients were classified as having high-output cardiac failure, of which three patients had a lower-arm AVF and seven an upper-arm AVF. These authors concluded that a Qa greater than 2000 ml/min reliably predicted the occurrence of high-output cardiac failure, since this was the case for all 10 patients with high-output cardiac failure. Recently, Zamboli et al. attempted to define the high-flow vascular access in 29 hemodialysis patients with a minimum Qa of 2000 ml/min, of which 8 had a lower-arm AVF and 21 had an upper-arm AVF.^26^ Patient characteristics, Qa and echocardiographic data were evaluated. They argued that the Qa rate should be corrected for the height^27^ of the patients and concluded that a Qa ⩾ 603 ml/min/m^27^ combined with echocardiographic alterations could identify patients at higher risk of high-output cardiac failure. Notably, none of these studies analyzed the association between Qa and cardiac function and/or outcome separately by type of vascular access. In the present study, the systolic and diastolic echocardiographic parameters did not differ significantly between the different vascular access groups. Thus, in patients with an upper-arm fistula, a similar degree of LVH and systolic and diastolic function was found compared with patients with a lower-arm fistula or a PTFE AVG, despite a significantly higher Qa.

Notably, both the LVMi and the prevalence of LVH were comparable for the different types of vascular access. This is remarkable given the significantly higher Qa in patients with an upper-arm AVF compared to those with a lower-arm AVF or PTFE AVG. These findings contrast with the results from previous studies that found a clear association between Qa and LVH.^27–29^ The importance of LVH is evident since it is important prognostic factor affecting survival of hemodialysis.^30^ Interestingly, we also found a reduced all-cause mortality in patients with an upper-arm AVF compared to those with a lower-arm AVF despite a much higher Qa in patients with a lower-arm AVF. This reduced all-cause mortality remained significant after adjustment for potential confounders including dialysis vintage and shunt vintage and even after additional adjustment for Qa. These contra-intuitive findings may be explained by divergent effects of an upper arm fistula versus a lower arm fistula on peripheral arterial resistance, blood pressure, and long-term cardiac workload. At the same time, we should be cautious with definite conclusions because this was an observational study with differences in patient characteristics between the lower-arm AVF group and the upper-arm AVF group. Despite our efforts to adjust for potential bias in our analyses, we cannot exclude the possibility of residual confounding.

Limitations of the current study are the relatively low number of patients, differences between groups and its cross-sectional design. Due to this cross-sectional design of this study, the interval between access creation and echocardiography was not standardized and this may potentially induce bias. However, inclusion of shunt vintage in the analysis of the associations between the type of vascular access and all-cause mortality did not change these associations. Moreover, shunt vintage was comparable in patients with an upper-arm AVF and lower-arm AVF. Therefore, varying intervals between access creation and the echocardiography have unlikely contributed to the observed differences in Qa and all-cause mortality between patients with an upper-arm and those with a lower-arm AVF. The original study focused on diastolic function parameters and regional wall motion abnormalities of the left ventricle. Unfortunately, reliable measurement of cardiac output was not available. However, other systolic cardiac function parameters like left ventricular ejection fraction (LVEF) and peak systolic velocity of mitral annular motion as determined by tissue Doppler (*Sʹ*) were available. Although all echocardiographic measurements were performed at a standardized time-point, it would have been informative to have follow-up measurements to monitor possible changes in Qa and cardiac function parameters over time. The strength of our study is that this is one of the largest studies on the association between type of access, cardiac function, and mortality. Furthermore, we provided detailed information on patient characteristics, vascular access characteristics, Qa, LVH, systolic and diastolic echocardiographic parameters, and long-term survival follow-up. Since this study is the first that compared echocardiographic parameters and outcome between patients with different types of vascular access, confirmation by other groups, is needed. Additionally, the possibly divergent effects of an upper arm fistula versus a lower arm fistula on peripheral arterial resistance, blood pressure, and long-term cardiac workload should be studied.

## Conclusion

Patients with an upper-arm AVF had comparable cardiac function parameters compared to patients with a lower-arm AVF and patients with a PTFE AVG and the lowest all-cause mortality, despite having the highest Qa. These findings should be confirmed in future research projects. It remains to be studied whether the favorable effects of an upper-arm fistula are caused by more favorable hemodynamic effects on the heart.

## References

[1] Vascular access work group. Clinical practice guidelines for vascular access. Am J Kidney Dis 2006; 48(Suppl 1): S248–S273. 16813991 10.1053/j.ajkd.2006.04.040

[2] Schmidli J Widmer MK Basile C , et al. Editor’s Choice – vascular access: 2018 clinical practice guidelines of the European Society for Vascular Surgery (ESVS). Eur J Vasc Endovasc Surg 2018; 55: 757–818. 29730128 10.1016/j.ejvs.2018.02.001

[3] Cheung AK Sarnak MJ Yan G , et al. Cardiac diseases in maintenance hemodialysis patients: results of the HEMO Study. Kidney Int 2004; 65: 2380–2389. 15149351 10.1111/j.1523-1755.2004.00657.x

[4] MacRae JM Pandeya S Humen DP , et al. Arteriovenous fistula-associated high-output cardiac failure: a review of mechanisms. Am J Kidney Dis 2004; 43: e17–e22. 15112194 10.1053/j.ajkd.2004.01.016

[5] Chemla ES Morsy M Anderson L , et al. Inflow reduction by distalization of anastomosis treats efficiently high-inflow high-cardiac output vascular access for hemodialysis. Semin Dial 2007; 20: 68–72. 17244125 10.1111/j.1525-139X.2007.00244.x

[6] Basile C Lomonte C Vernaglione L , et al. The relationship between the flow of arteriovenous fistula and cardiac output in haemodialysis patients. Nephrol Dial Transplant 2008; 23: 282–287. 17942475 10.1093/ndt/gfm549

[7] Basile C Vernaglione L Casucci F , et al. The impact of haemodialysis arteriovenous fistula on haemodynamic parameters of the cardiovascular system. Clin Kidney J 2016; 9: 729–734. 27679720 10.1093/ckj/sfw063 PMC5036899

[8] Di Lullo L Floccari F Polito P . Right ventricular diastolic function in dialysis patients could be affected by vascular access. Nephron Clin Pract 2011; 118: c257–c261. 21196771 10.1159/000321867

[9] Dundon BK Torpey K Nelson AJ , et al. The deleterious effects of arteriovenous fistula-creation on the cardiovascular system: a longitudinal magnetic resonance imaging study. Int J Nephrol Renovasc Dis 2014; 7: 337–345. 25258554 10.2147/IJNRD.S66390 PMC4172192

[10] Assa S Hummel YM Voors AA , et al. Changes in left ventricular diastolic function during hemodialysis sessions. Am J Kidney Dis 2013; 62: 549–556. 23548554 10.1053/j.ajkd.2013.02.356

[11] Loutradis C Sarafidis PA Papadopoulos CE , et al. The Ebb and flow of echocardiographic cardiac function parameters in relationship to hemodialysis treatment in patients with ESRD. J Am Soc Nephrol 2018; 29: 1372–1381. 29592914 10.1681/ASN.2017101102 PMC5967760

[12] Pandeya S Lindsay RM . The relationship between cardiac output and access flow during hemodialysis. ASAIO J 1999; 45: 135–138. 10360711 10.1097/00002480-199905000-00006

[13] Ahearn DJ Maher JF . Heart failure as a complication of hemodialysis arteriovenous fistula. Ann Intern Med 1972; 77: 201–204. 4641654 10.7326/0003-4819-77-2-201

[14] Anderson CB Codd JR Graff RA , et al. Cardiac failure and upper extremity arteriovenous dialysis fistulas: case reports and a review of the literature. Arch Intern Med 1976; 136: 292–297. 1259499

[15] Engelberts I Tordoir JH Boon ES , et al. High-output cardiac failure due to excessive shunting in a hemodialysis access fistula: an easily overlooked diagnosis. Am J Nephrol 1995; 15: 323–326. 7573191 10.1159/000168857

[16] Young PR Rohr MS Marterre WF . High-output cardiac failure secondary to a brachiocephalic arteriovenous hemodialysis fistula: two cases. Am Surg 1998; 64: 239–241. 9520814

[17] Bailey WB Talley JD . High-output cardiac failure related to hemodialysis arteriovenous fistula. J Ark Med Soc 2000; 96: 340–341. 10705682

[18] Trespalacios FC Taylor AJ Agodoa LY , et al. Heart failure as a cause for hospitalization in chronic dialysis patients. Am J Kidney Dis 2003; 41: 1267–1277. 12776280 10.1016/s0272-6386(03)00359-7

[19] Murray BM Rajczak S Herman A , et al. Effect of surgical banding of a high-flow fistula on access flow and cardiac output: intraoperative and long-term measurements. Am J Kidney Dis 2004; 44: 1090–1096. 15558531 10.1053/j.ajkd.2004.06.031

[20] Assa S Hummel YM Voors AA , et al. Hemodialysis-induced regional left ventricular systolic dysfunction and inflammation: a cross-sectional study. Am J Kidney Dis 2014; 64: 265–273. 24364893 10.1053/j.ajkd.2013.11.010

[21] Nagueh SF Appleton CP Gillebert TC , et al. Recommendations for the evaluation of left ventricular diastolic function by echocardiography. Eur J Echocardiogr 2009; 10: 165–193. 10.1093/ejechocard/jep007 19270053

[22] Devereux RB Alonso DR Lutas EM , et al. Echocardiographic assessment of left ventricular hypertrophy: comparison to necropsy findings. Am J Cardiol 1986; 57: 450–458. 2936235 10.1016/0002-9149(86)90771-x

[23] Lang RM Badano LP Mor-Avi V , et al. Recommendations for cardiac chamber quantification by echocardiography in adults: an update from the American Society of Echocardiography and the European Association of Cardiovascular Imaging. J Am Soc Echocardiogr 2015; 28: 1–39.e14. 10.1016/j.echo.2014.10.003 25559473

[24] Sterne JA Carlin JB Spratt M , et al. Multiple imputation for missing data in epidemiological and clinical research: potential and pitfalls. BMJ 2009; 338: b2393. 19564179 10.1136/bmj.b2393 PMC2714692

[25] Harel O Zhou XH . Multiple imputation: review of theory, implementation and software. Stat Med 2007; 26: 3057–3077. 17256804 10.1002/sim.2787

[26] Zamboli P Luca S Borrelli S , et al. High-flow arteriovenous fistula and heart failure: could the indexation of blood flow rate and echocardiography have a role in the identification of patients at higher risk? J Nephrol 2018; 31: 975–983. 29357085 10.1007/s40620-018-0472-8

[27] Valerianova A Malik J Janeckova J , et al. Reduction of arteriovenous access blood flow leads to biventricular unloading in haemodialysis patients. Int J Cardiol 2021; 334: 148–153. 33895210 10.1016/j.ijcard.2021.04.027

[28] Zheng H Bu S Song Y , et al. To ligate or not to ligate: a meta-analysis of cardiac effects and allograft function following arteriovenous fistula closure in renal transplant recipients. Ann Vasc Surg 2020; 63: 287–292. 31536798 10.1016/j.avsg.2019.06.040

[29] Malik J Lomonte C Rotmans J , et al. Hemodialysis vascular access affects heart function and outcomes: tips for choosing the right access for the individual patient. J Vasc Access 2021; 22: 32–41. 33143540 10.1177/1129729820969314 PMC8606800

[30] Silberberg JS Barre PE Prichard SS , et al. Impact of left ventricular hypertrophy on survival in end-stage renal disease. Kidney Int 1989; 36: 286–290. 2528654 10.1038/ki.1989.192

